# Challenges and solutions to talent (de)selection and development in a youth soccer academy: the implementation of a multidisciplinary athlete profiling tool

**DOI:** 10.3389/fpsyg.2025.1636386

**Published:** 2025-11-04

**Authors:** Sam Barraclough, Kevin Till, Adam Kerr, Stacey Emmonds

**Affiliations:** ^1^Department of Sport and Exercise Sciences, Manchester Metropolitan University, Manchester, United Kingdom; ^2^Carnegie School of Sport, Leeds Beckett University, Leeds, United Kingdom; ^3^Leeds United Football Club, Leeds, United Kingdom

**Keywords:** talent identification, youth, soccer, multidisciplinary, selection, profiling, challenges

## Abstract

Youth male soccer is more competitive than ever as clubs strive to recruit, develop, and produce future elite soccer players. Within youth soccer academies, it is important to recognise that, talent development (TD) and (de)selection are on-going and complex multidisciplinary processes with several challenges. Part 1 of this paper presents three real-world practical challenges including: (1) the ability to differentiate between performance and potential, (2) understanding and alignment to develop talented players, and (3) decision-making processes and (de)selection of players. The paper then presents a possible solution to these challenges demonstrated through the application of a multidisciplinary profiling tool created and utilised within a youth male soccer academy in the UK (Part 2). Finally, Part 3 of the paper identifies the applied challenges associated with implementing such a tool within a TD environment and provides possible solutions for effective implementation. The creation and implementation of the multidisciplinary athlete profiling tool can facilitate TD, and support (de)selection decisions. The solutions provided may serve as principles that can be flexibly implemented across a variety of sports and environments to augment TD and (de)selection processes.

## Introduction

Recognising, developing, and (de)selecting talented youth athletes is a complex and multifactorial process. These processes are often ‘coach driven’, whereby coaches aim to decide and predict, often through their own intuition or instinct (Christensen, 2009; Bergkamp et al., 2021; Roberts et al., 2021), a young athlete’s needs and their potential for future performance success. During talent development (TD), defined as a systematic programme of coaching, support, training, and competition designed to progress players (Williams et al., 2020), periods of (de)selection occur at frequent timepoints (Ford et al., 2020). This (de)selection procedure is defined as an on-going process of choosing players within the development programme who are (selection) or are not (deselection) exhibiting the necessary attributes deemed to be suitable for progression to a future squad or team (Williams et al., 2020).

Within TD and (de)selection processes, previous research has suggested coaches often aim to observe recognisable combinations of various attributes (e.g., high motivation, technical proficiency, sprint speed) that previous experience has shown them are suggestive of future performance levels (Christensen, 2009; Johansson and Fahlén, 2017; Roberts et al., 2019a). The recognition of these attributes is said to stem from a coach’s tacit knowledge and expertise (i.e., their subjective expert opinion; SEO), formed into a socially constructed image of what a talented athlete should be (Roberts et al., 2019a; Bergkamp et al., 2022). However, this often causes inconsistencies and biases in appraisals of an athlete’s current performance and their potential for future success (Baker et al., 2018). Such biases may be particularly problematic in the case of youth soccer academies, as TD and (de)selection decisions are typically conducted in a multidisciplinary team (MDT) environment, requiring active cooperation and collaboration across multiple staff (Reid et al., 2004; Salcinovic et al., 2022). In such scenarios, any conflict or disagreement (possibly due to individual biases) may result in less accurate TD plans, or in harsher circumstances, a player being (wrongly) deselected from a programme.

Previous research has established possible issues relating to TD and (de)selection processes (Baker et al., 2018; Johnston et al., 2018; Till and Baker, 2020), with further research providing examples of several theoretical issues (Bergkamp et al., 2019; Johnston and Baker, 2020; Barraclough et al., 2022). Together, these works and others, substantiate that the quality of evidence utilised during talent decision-making may be conceptually questionable. For example, Johnston and Baker (2020), highlighted that TD and (de)selection decisions can be inaccurate, biased, and illogical, contributing to talent being ‘wasted’. However, limited literature explores solutions-based thinking to these issues in applied practice (Eisenmann et al., 2020; Webdale et al., 2020; Till et al., 2023; Kelly et al., 2025). Therefore, Part 1 of this paper highlights real-world practical challenges to TD and (de)selection within academy soccer. Part 2 then presents possible solutions to these challenges through a real-world example of designing and applying a multidisciplinary athlete profiling tool within a youth soccer academy. Part 3 then summarises the challenges associated with implementing an athlete profiling tool and solutions to driving its success.

## Part 1: challenges to talent development and (de)selection

### Challenge 1: Recognising talent—differentiating between performance and potential

Research highlights that there is a lack of consensus regarding definitions of talent, e.g., (Baker et al., 2019; Issurin, 2017; Simonton, 1999), creating a vague and “blurry” term that lacks conceptual clarity (Johnston et al., 2023). With the concept of talent being ill-defined and poorly understood (Grainger et al., 2024), questions have been raised regarding its utility, including suggestions to retire such terminology from applied environments (Baker et al., 2024). Given the complexities surrounding the concept of talent, performance and potential have emerged as two key factors to aid practitioners in their decision-making processes, and considerations of how they view talent (Baker et al., 2018; Till and Baker, 2020). Performance and potential ratings have been applied within youth soccer academies, whereby coaches rated athletes’ performances on a weekly basis (e.g., Hill et al., 2020; Kite et al., 2023) and potential on a 6-weekly/quarterly basis (e.g., Barraclough et al., 2024b; Kite et al., 2023). However, these ratings can still be problematic for coaches, who attempt to identify potential without objective or valid measures of its existence (Baker et al., 2019). This leads to difficulties in differentiating current performance and potential (Baker et al., 2018; Barraclough et al., 2024b).

A clear distinction between performance and potential has been found within some research (Barth et al., 2022; Herrebrøden and Bjørndal, 2022; Güllich et al., 2023). For example, Güllich et al. (2023) showed international youth athletes failed to reach senior international level highlighting that the performance level of youth ‘talented’ athletes does not always equate to future success (Barreiros et al., 2014). Furthermore, research has shown maturity-related selection biases within youth soccer, with a tendency for selection and retention of athletes more advanced in biological maturity (Hill et al., 2023), where advanced biological age in an athlete significantly predicted a higher perceived level of performance (Hill et al., 2020). These findings highlight the confounding effect of biological maturation upon distinguishing athlete performance and potential. Additionally, factors such as sustaining an injury (Bangert et al., 2024), and current psychosocial skills (Gledhill et al., 2017) may also be detrimental when attempting to differentiate performance and potential.

In summary, there appears to be a common misconception within youth sport surrounding the concept of talent and the differences between performance and potential. This presents an on-going challenge for coaches and practitioners within applied practice, who attempt to make inferences about the potential for future success based on an athlete’s current performance (Barraclough et al., 2024b). This is further compounded by the effects of biological maturation (Hill et al., 2020, 2023). In such cases, coaches and practitioners must endeavour to mitigate (sub)conscious biases in their player evaluations.

### Challenge 2: Evaluating performance—a lack of agreement

Whilst the concepts of talent and potential are challenging, assessing an athlete’s current level of performance is viewed as an achievable task by scouts and coaches, who often assess performance based on their instinct and previous experiences (Roberts et al., 2019a; Bergkamp et al., 2021, 2022). However, differing opinions of athletes between staff makes consistent decision-making challenging - due to individual preferences of athletes and/or their attributes, or an athlete’s suitability for a particular playing style or philosophy (Christensen, 2009; Johnston and Baker, 2020). Several sub(conscious) biases exist [e.g., maturity biases; Hill et al. (2021), Sweeney et al. (2023), confirmation bias; Kite et al. (2023), the endowment effect; Johnston and Baker (2020), relative age affects; Hill et al. (2021) and Leyhr et al. (2021)] presenting an inherent challenge within youth soccer organisations; whereby key staff demonstrate a lack of agreement when assessing performance (Bergkamp et al., 2022; Lüdin et al., 2023).

An existing body of evidence highlights the differences in attributes that coaches and scouts deem important (e.g., Bergkamp et al., 2021; Kite et al., 2021; Roberts et al., 2019b; Towlson et al., 2019). For example, Bergkamp et al. (2021) showed that 125 scouts from the Netherlands considered technical attributes as the best predictors for future performance, whereas in the UK, Kite et al. (2021) identified that psychological attributes were perceived as most important amongst 30 academy staff in relation to talent and development. Whilst this conflict in findings may be representative of cultural and individual preferences (e.g., potential differences in playing styles; Plakias et al., 2023) there is still an absence of research demonstrating a unified approach to identifying and developing talent. Through the operationalization and subsequent scoring of specific criteria [i.e., key performance indicators; KPI’s; Hendry et al., 2018 and Unnithan et al. (2012)], coaches, scouts, and practitioners may be afforded an opportunity to provide more reliable and unbiased assessments. However, research that has attempted to reduce biases in the assessment of athlete performances has: (i) failed to specify the criteria on which assessments are based (Zuber and Conzelmann, 2014), (ii) highlighted the difficulty in utilising a large number of criteria (Reeves et al., 2019), or (iii) provided minimal detail regarding the conceptualization or determination of such criteria (Fenner et al., 2016; Höner et al., 2021), and (iv) has demonstrated a lack of agreement between coaches (Wiemeyer, 2003).

Given the plethora of individual preferences and subjective opinions in relation to an athlete’s performance, completely eliminating bias is an unattainable task. However, understanding, agreeing upon, and operationalising the use of an explicit collection of criteria when assessing an athlete, may provide a more consistent framework for developing agreement between practitioners on athlete performance.

### Challenge 3: Athlete development—assessing multidisciplinary change

Talent is defined as a multi-dimensional, emergenic, and dynamic concept (Baker et al., 2019). Therefore, TD should not be viewed as a static process and the multidisciplinary nature of soccer performance itself means (e.g., psychological traits, physiological attributes, technical skills, tactical awareness). Yet to date, most research has been described as adopting a unidimensional approach (Baker et al., 2020; Barraclough et al., 2022). Additionally, without tracking the progression, development, and changes in a multidisciplinary athlete profile over time (i.e., longitudinal monitoring) any information derived from an athlete profile is at risk of being misinterpreted and/or utilised out of context. For example, taking a reductionist approach and basing (de)selection decisions solely on the importance of physical attribures of players at pre-pubertal age groups (e.g., Fortin-Guichard et al., 2022; Gil et al., 2014) without clear indication of how these attributes may develop in the future (Moran et al., 2020). This raises several ethical concerns in relation to the collection and (mis)use of athlete profiling data. Furthermore, soccer performance emerges from complex interactions, between multiple athletes, in response to tasks, which occur in a dynamic and varying relationship within the environment (Araújo et al., 2006). Therefore, there is a need to not only subjectively assess soccer performance in isolation (e.g., through coach/scout SEO), but to also assess data that represents the underpinning attributes that contribute to performance [e.g., signs; Bergkamp et al. (2019) and Den Hartigh et al. (2018)] and data that contextualises the events required for successful performance [e.g., samples; Barraclough et al. (2022) and Bergkamp et al. (2019)].

With this in mind, it is recommended that decision-making on any athlete should not be exclusively based on any single characteristic in isolation. The combination of objective and subjective multidisciplinary data is crucial in providing challenge or support to any subjective opinions and may fuel important discussion around athlete needs and development plans (McIntosh et al., 2019; Dugdale et al., 2020; McCormack et al., 2022).

## Part 2: Possible solutions—an applied example from an elite youth male soccer academy

To address the challenges discussed above, several possible solutions can be implemented in practice. This section reviews these possible solutions before presenting an applied example that has been designed and implemented within a football academy in the UK.

### Performance and potential

A player exhibiting high performance and showing improvement in multiple areas (e.g., physical, technical, psychosocial, and tactical development) may in fact be demonstrating high potential, evidenced through their ability to frequently demonstrate the necessary characteristics to be retained and progress within their organisation. Monitoring an athlete’s development over time, can prompt a recurring yet necessary message within youth coaches—the value of long-term development over short-term results (Martindale et al., 2007). As outlined in challenge 1, coaches working with youth athletes should have a level of clarity in their understanding of (de)selection and TD, recognising and acknowledging that success at an early age is not always a pre-requisite of the potential for future success (e.g., Güllich et al., 2023). Further, coaches should be aware of the non- linear development of youth athletes (Abbott et al., 2005), and view talent not as a static quality related to current performance, but as a highly individual, emergent property heavily influenced by the environment (Baker et al., 2019).

### Growth and maturity considerations

To discern the differences between performance and potential, consideration of individual growth and maturity data should be considered. This provides an appropriate lens through which to view an athlete’s current performance and potential and may challenge one of the most common selection biases within youth soccer [i.e., maturity selection biases; Hill et al. (2021) and Sweeney et al. (2023)]. Referencing of objective data against biologically derived benchmarks (Till and Jones, 2015; Till et al., 2018) or use of subjective performance ratings utilising bio-banded training sessions or matches (Cumming et al., 2017; Abbott et al., 2019; Bradley et al., 2019) can change a coach’s perspective of an athlete’s ability through comparisons with more heterogenous peers. For example, research suggests that bio-banded competition changes the technical demands placed upon youth athletes compared to chronological competition, with a reduction or maintenance in the physical demands (e.g., Cumming et al., 2018; Abbott et al., 2019). Therefore, such comparisons permit fairer observation of an athlete’s performance in the context of their individual stage of growth and maturity and allows the creation of more appropriate developmental training plans (i.e., TD). For example, reducing training loads to mitigate injury risk around rapid periods of growth (Johnson et al., 2023).

### A multidisciplinary approach

The idea of what a talented athlete is will vary amongst coaches based on their own preferences and experiences, with research suggesting coaches are unable to agree upon or articulate the specific combinations of attributes they are searching for when observing talented athletes (Wiseman et al., 2014; Roberts et al., 2019a). Whilst a coaches’ ability to predict future performance has shown high accuracy in sports such as swimming (Crowcroft et al., 2020), predicting potential in a team sport such as soccer, which is not defined by a single competition result or skill (e.g., swim time), may be more challenging. Soccer performance emerges in a dynamic and varying environment, performing skills underpinned by complex interactions of different attributes across multiple disciplines [e.g., physiological, psychological, technical, tactical, sociological; Stølen et al. (2005)]. Therefore, a multidisciplinary approach considering information and data from a variety of sources may be considered best practice in TD and (de)selection processes, providing stakeholders are transparent with players and their parents/guardians in relation to obtaining informed consent for collection and use of such data.

### A shared mental model of performance

By agreeing upon, defining, and understanding what successful performance entails (within the context of an organisation), coaches thinking can be formed into a shared mental model [SMM; Barraclough et al. (2024a)]. Such a model can help to co-ordinate coaches’ and scouts’ actions and decision-making, providing a shared and coherent framework to promote alignment in organisational thinking (Price and Collins, 2022), which may attenuate subjective biases in decision-making. This theoretically reduces the variability of opinions amongst coaching and recruitment staff, leading to a decrease in disparate assessments of athlete performances and an aligned approach to TD and (de)selection. By explicitly defining key position specific actions that contribute to successful performance, a SMM can be shaped that embodies the dynamic and complex nature of soccer and encapsulates many of the underpinning multidisciplinary attributes coaches may deem important (Roberts et al., 2019a; Kite et al., 2021; Barraclough et al., 2024a) This SMM can enhance the creation of a coherent pathway within TD environments (Martindale et al., 2007; Webb et al., 2016), through providing greater understanding of what “talent” looks like within that organisation. Equally, the differing long-term trajectories of TD should also be accounted for within the SMM, ensuring that a level of variability is accounted for based on varying rates of individual development (Cobley et al., 2014; Webb et al., 2016).

### Signs, samples, and SEO

Whilst coaches view their SEO as a legitimate method for informing decision-making around TD and (de)selection, the validity and reliability of these highly subjective decisions has been called into question (Johansson and Fahlén, 2017; Bergkamp et al., 2022). As such, a more comprehensive approach should combine objective and subjective data based on an athlete’s ability to perform the soccer-specific actions required for successful performance (Barraclough et al., 2025). Profiling data provides objective isolated assessments (signs) of the underlying attributes (physical, psychological, technical, and tactical) that contribute to performing a specific skill or action and provides context around if an athlete ‘can’ perform the skills required. Equally, training and match performance analysis data (samples) offer authentic, contextually rich, and objective insights into athletes’ performance of soccer-specific actions, thereby enhancing understanding of whether players ‘are’ performing the requisite skills – skills that have been empirically linked to increased likelihood of match success (e.g., Fernández-Cortés et al., 2023). Finally, a coach’s SEO may be used to judge the ‘impact’ a player is having through a subjective scoring of an athlete’s overall match performance. This permits a comprehensive framework for interpreting an athlete’s skills and abilities and can facilitate a comprehensive understanding of an athlete’s current level of performance. Notably, the addition of sources of unbiased objective data (signs and samples), utilised in conjunction with subjective expertise (SEO) permits the checking and challenging of staff’s opinions on players and can guide MDT discussions.

### Longitudinal monitoring

Given the primary objective of a soccer TD environment is to nurture and develop talented young players (Relvas et al., 2010; Williams et al., 2020), information to facilitate and evaluate such development should stem from longitudinal analysis of a player’s strengths and weaknesses utilising the possible solutions previously raised. Research has shown that many of the associated qualities linked with successful adult performance are not established within individuals prior to late adolescence and a near adult level of biological and psychosocial maturity (Fransen et al., 2017; Eisenmann et al., 2020). Consequently, the use of cross-sectional information and the efficacy of performance or attributes at young ages as predictors of elite adult success are contentious. Through longitudinal monitoring and retrospective analysis, the non-linear development of those who succeed within TD environments and achieve professional status and success can be highlighted. Additionally, despite contrasting evidence on rates of longitudinal physical development between future professional and non-professional players within the literature (e.g., Roescher et al., 2010; Saward et al., 2016; Leyhr et al., 2018; Fortin-Guichard et al., 2022), observation of specific trends and changes in physical (and other areas of performance) may be indicative of an athlete’s future potential (e.g., consistent development above and beyond normal expectations), providing further information and utility for TD and (de)selection purposes.

### An applied example from an elite youth male soccer academy

Having highlighted some real-world challenges and possible solutions surrounding the recognition, development, and (de)selection of talent, this paper now presents and discusses an applied example of incorporating a multidisciplinary, longitudinal athlete profiling tool within the context of a youth male soccer academy in the UK. This tool was implemented in a real-world context aligning with calls for more practical and action-oriented research in TD (Collins et al., 2019). It draws upon the possible solutions highlighted above, to address the challenges presented in Part 1. The information provided within the tool has been suggested previously (e.g., Kelly et al., 2018) and discussed in the context of other European (e.g., Lavandera, 1999) soccer academies. Such information is commonly collected and utilised as part of TD and (de)selection processes in academy soccer. Specifically, the tool was created to support TD and (de)selection of athletes through: (i) considerations of performance and potential, (ii) use of a shared mental model of performance (iii) collecting and visualising multidisciplinary data, (iv) using signs, samples and SEO data, (v) demonstrating varying growth and maturity of athletes, and (vi) collecting data longitudinally.

**Figure 1:**
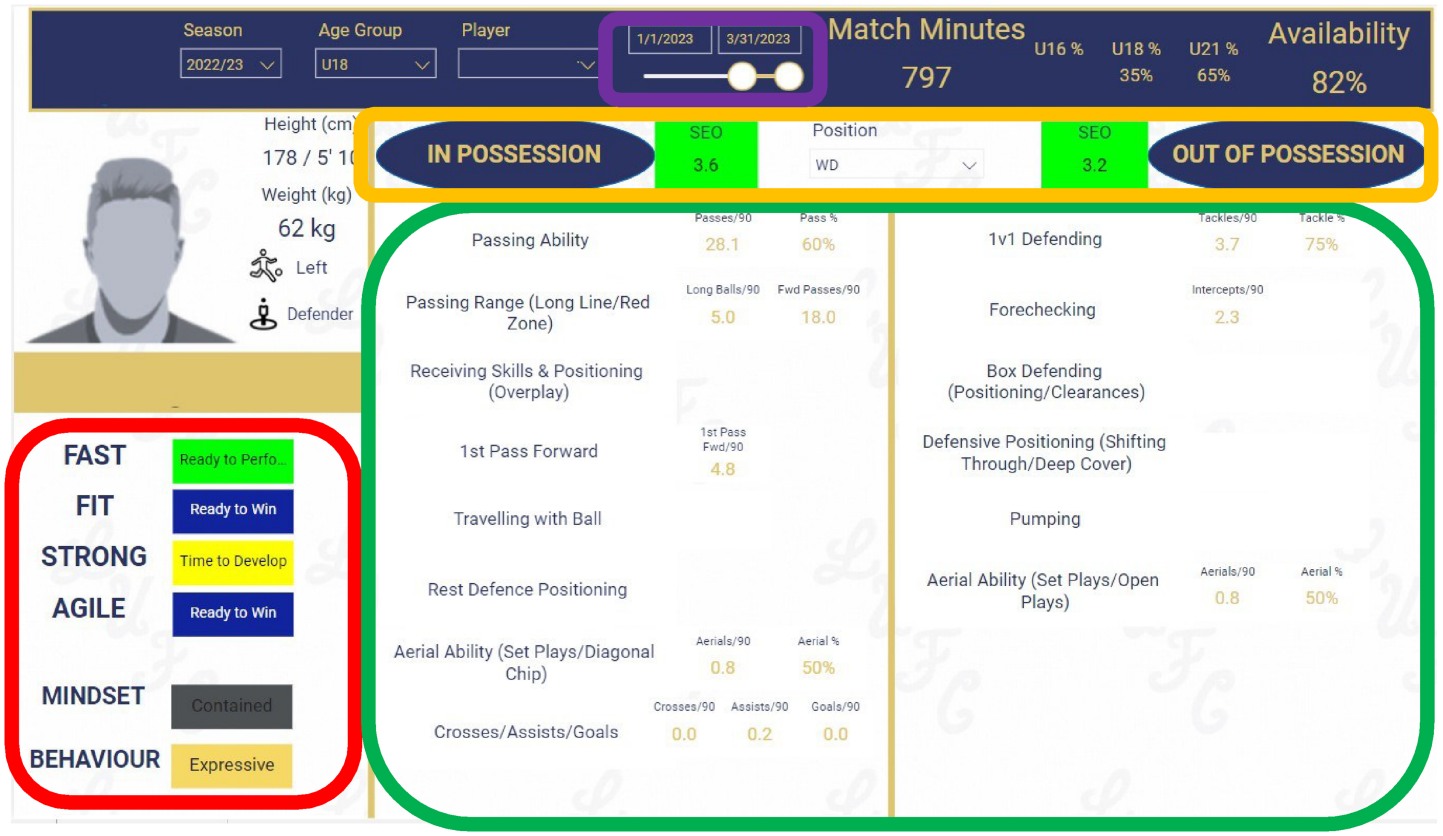
Athlete profiling tool using a multidisciplinary, objective and subjective dataset consisting of signs, samples, and subjective expert opinion with longitudinal date filter.

This tool used an interactive format to visualise data from a variety of sources through dashboards and reports (Microsoft Power Bi) and was embedded within the academy’s shared cloud-based, team collaboration platform (Microsoft Teams). This made it easily accessible and interpretable for users and provided them with insights on individual athletes. Figure 1 presents an example report from within the multidisciplinary athlete profiling tool, summarising key information for an individual athlete, which would form the basis of MDT discussions for each athlete. Underpinning attributes deemed important for performance (signs) are highlighted in the red box demonstrating the specific athlete’s physical and psychological profiling data. For the physical profiling, a composite score [Total Score of Athleticism (TSA); Turner et al. (2019)] from physical tests mandated by the elite player performance plan [EPPP; The Premier League (2011)], and with previously proven predictive validity (Murr et al., 2018; Kelly and Williams, 2020), was calculated and a colour coded graded system based on individual growth and maturation was used to represent each athlete. Psychological profiling demonstrated an athlete’s psychological and behavioural adaptability through identifying their personality strengths from a mindset and behavioural perspective. Samples (performance analysis statistics) of performance (across multiple matches) which represent actions from the SMM are highlighted in the green box with the associated score representing the athlete’s mean across the matches for the period chosen. Equally, a mean score for the coach’s SEO of performance across those matches is highlighted in the yellow box. Mean scores were utilised as they represented general performance over multiple matches, discounting possible one-off or atypical performances from individual players. The adjustable date filter highlighted in the purple box provided an adjustable date range permitting variable longitudinal evaluation (e.g., 6-weekly, quarterly, yearly).

## Part 3: Challenges and solutions to implementing a multidisciplinary athlete profiling tool

Whilst the design of the athlete profiling tool was derived from research and principles relating to talent identification (TID), (de)selection, and TD, further challenges for the implementation of such a system exist in practice. The final part of this manuscript summarises the challenges and solutions to implementing, using, and maximising such a tool within practice, within the context of a youth male football academy in the UK. Importantly, the implementation of the tool aimed to enhance the quality of evidence available for TD and (de)selection decisions through providing longitudinal, multidisciplinary objective and subjective data, leading to decisions being based on more complete and consistent evidence.

### Challenge 1: Data processing

The creation of such a tool requires the seamless collection, management, analysis, and reporting of multiple sources of data within the day-to-day constraints of the TD environment. One of the difficulties in the first stage of this process is the variety of data collection sources and adherence to guiding scientific principles that ensure collection of accurate, valid, reliable, and sensitive data (Currell and Jeukendrup, 2008). Further, given the volume of data being collected, data collection procedures need to be as efficient as possible to minimise the time-loss burden within a fast-moving environment. In addition to the standardisation of data collection procedures to increase validity, the reliability of data capture should also be considered. For example, considering the level of agreement made by different observers when subjectively scoring (e.g., Bergkamp et al., 2022; Lüdin et al., 2023) or quantifying technical and tactical performance measures (e.g., Liu et al., 2013). Therefore, challenges exist in creating data collection procedures that maximise practical utility (i.e., procedures that are, where possible, uncomplicated, transparent, and accomplishable by numerous members of staff). Finally, informed consent must be obtained for collecting and processing such data, in addition to ensuring secure storage and access in line with data privacy regulations (e.g., General Data Protection Regulation; GDPR).

Challenges also exist in the subsequent stages of data collection process. For example, once data capture is complete it is vital that care is taken in data entry and management procedures to reduce the risk of data errors - as these errors can potentially have a large impact on the interpretation of results (Barchard and Pace, 2011). Additionally, within team sports such as soccer, considerable volumes of data are often collected simultaneously from multiple sources (e.g., an entire team undertaking a complete fitness testing battery, match analysis statistics for each player, subjective scoring). A challenge exists in the requirement to centralise these large volumes of data, often originating from multiple external data providers. This typically requires access to appropriate software to enable extraction, transformation, and loading (ETL) of the data, as well as the ability to securely store data and create appropriate relationships between data sources.

Finally, analysis and reporting/visualisation of the data presents a challenge in its own right, encompassing the need to maximise information concisely. The challenge presented here is the need to simplify potentially complex information and portray it in an aesthetically pleasing format to facilitate interpretation (Nosek et al., 2021). Without such an approach for easily accessible and interpretable reporting, the need for answers can often lead to hasty decision-making that fails to consider potentially relevant information. In summary, there is a challenge for data to be managed efficiently, analysed, reported, and shared across multiple users. Through an efficient data process, the information provided can serve its intended purpose and be utilised as a support system that augments decision-making regarding (de)selection and TD (Robertson et al., 2017).

### Solution 1

#### Streamlined data processes

**Figure 2:**
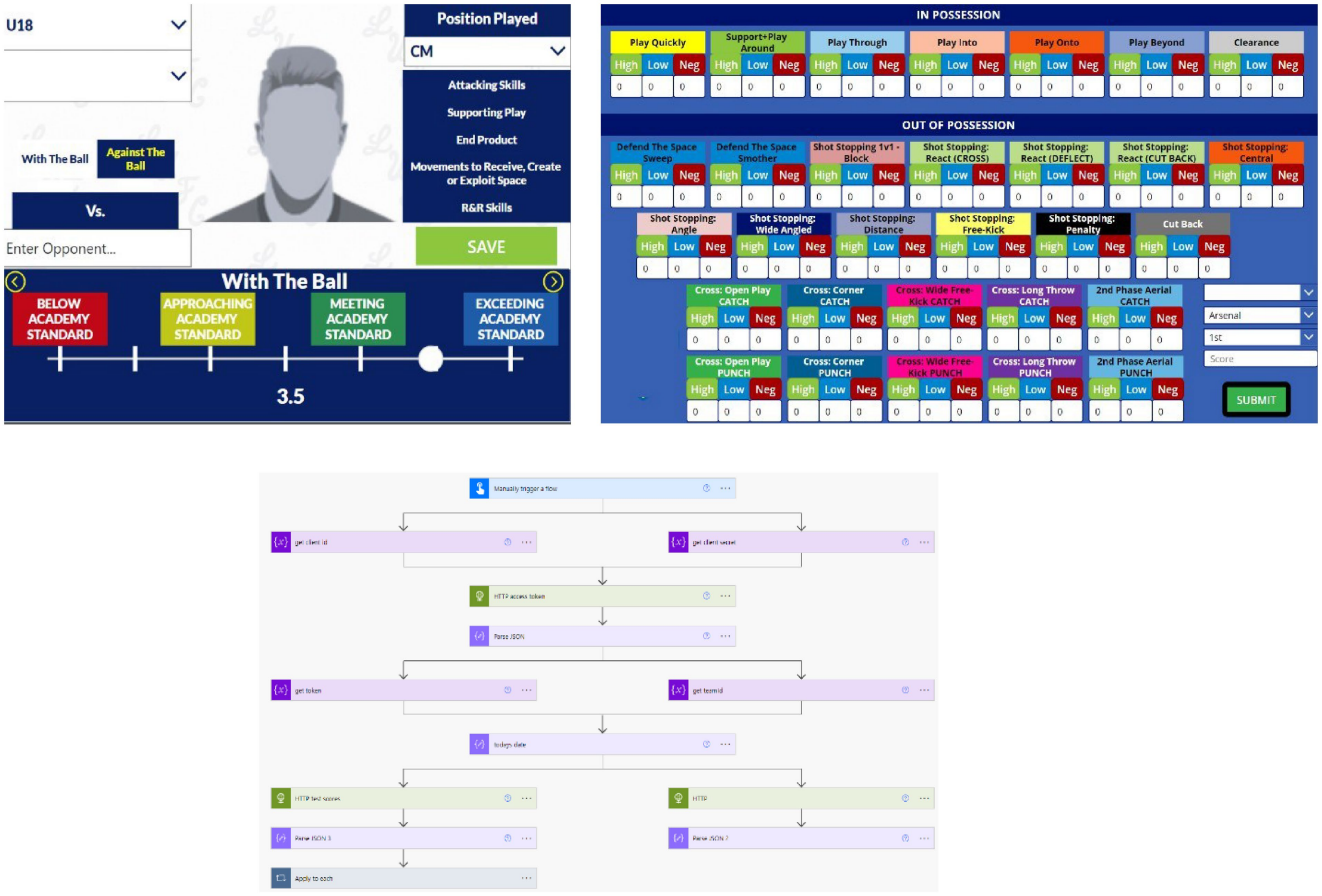
Example use of various Microsoft software utilised in the data process within the organisation. **(A)** Microsoft Power Apps to create an internal app for player ratings. **(B)** Microsoft Power Apps GK performance analysis coding window. **(C)** Microsoft Power Automate flow demonstrating API calls to various software providers returning relevant data for central storage.

One solution to overcome challenges associated with data collection is the use of valid, reliable, and sensitive measurements of various aspects of performance, and their associated protocols (e.g., the creation of a physical profiling handbook/instruction manual). Secondly, given the rise in technology and the use of cloud-based infrastructure, relevant software (e.g., Microsoft Office, Microsoft Teams, and Microsoft Power Platform) can be used to maximise the efficiency of securely managing, storing, and sharing organisational data for the tool, following receipt of informed consent for individual players. Such software also allows the integration of application programming interfaces (API’s) and applications (apps) to collect, integrate and share information. Figure 2 provides example usage of such software demonstrating – (a) an app for coaches/scouts to provide their SEO of match performance for individual players based upon soccer-specific actions from a SMM, (b) a customised coding window to collect samples data (match analysis statistics), and (c) an API flow diagram to collect and centrally store data from various sources.

**Figure 3:**
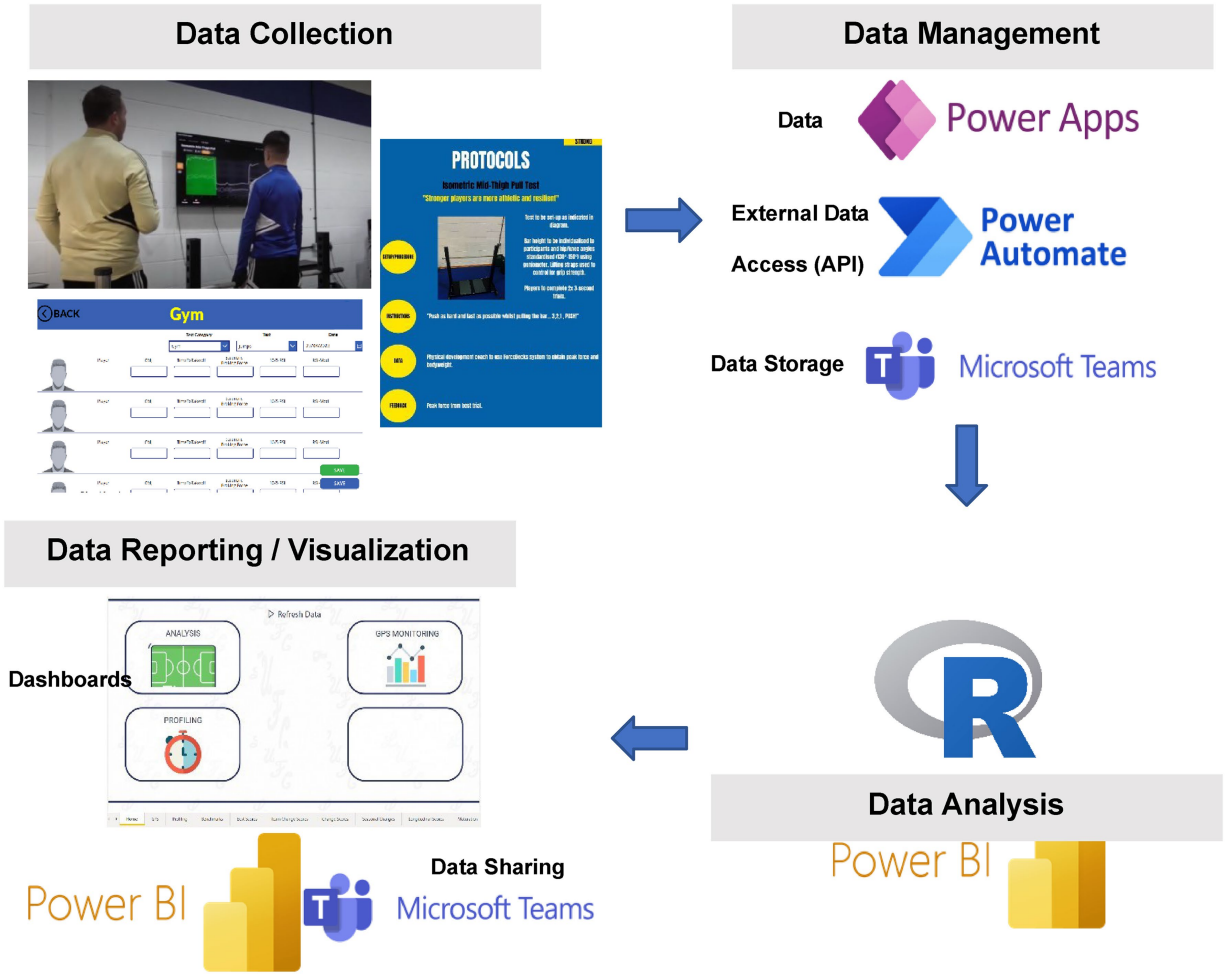
Data process within the organisation consisting of data collection, data management, data analysis and data reporting/visualization.

The integration of such software can streamline data management processes through assembling data from a variety of sources, creating relationships between data sources, and forming a relational data model. Use of these software to collect, manage, analyse, and feedback/visualise data allows knowledge to be shared across multiple staff within the organisation. This prompts opportunities for timely and relevant conversations that can act to support the decision-making process regarding an individual athlete’s development. Figure 3 provides a schematic overview of the data processing sequence showing data collection (apps and physical profiling handbook), data management (software used to collect, transform, and store the data), data analysis (software used to clean and analyse the data), data reporting/visualisation (software used to embed the tool within practice).

### Challenge 2: MDT engagement and decision-making

When considering the use of the tool for an individual athlete, it is vital for key stakeholders to engage with the information being provided as a platform to discuss an appropriate development plan (i.e., TD) for the athlete. The challenge presented here relates to decision-making within the MDT and the effective use and interpretation of the data provided. The example tool, providing the multidisciplinary profile and its related data, should be utilised to help support the decision-making process, a process that should be data-informed rather than data driven (Gamble et al., 2020). Whilst implementation of a comprehensive athlete profiling tool can positively facilitate (de)selection, and TD processes – there is a requirement for all MDT staff to engage with and have confidence in the information at their disposal. Part of this engagement may however be demonstrated on a longer-term basis, through reflections on the use of the tool to gauge its effectiveness (i.e., its efficacy in the development and selection of successful athletes).

### Solution 2

#### Data-informed, multidisciplinary decision-making

An MDT meeting and collective decision-making process provides a natural opportunity to maximise the interpretation of an athlete’s profile through conversations with all relevant staff. A data-informed approach allows individual staff to engage in the process and provide their own interpretations of the data, aligned with their expertise. This can add context to the data provided within the tool, promoting discussion amongst the various staff members. This can guide the MDT process and direct staff towards the appropriate conversations (i.e., what actions can / cannot a player perform successfully). Once an understanding of an athlete’s strengths and weaknesses in relation to their performances have been identified and agreed upon, appropriate conversations within the MDT can take place allowing collective decisions to be made regarding (de)selection, and TD.

Finally, a thorough review of the TD programme and (de)selection decisions at specific timepoints throughout and across seasons allows critical reflection on the effectiveness of such a tool and the programme as a whole. Reflecting on the programme and use of the tool may highlight necessary changes based on both positive and negative outcomes as a result of the MDT decision-making process and can help facilitate future planning. A consistent and recurring process can then provide key examples of previous successes that may be considered best-practice to help inform future decision-making.

## Limitations and future directions

This study presents the design and implementation of a multidisciplinary athlete profiling tool within a single UK youth male soccer academy. While the theoretical principles underpinning the tool are informed by current literature and are widely applicable across other TD structures, the theoretical solutions should be interpreted with caution. Firstly, whilst organisations globally may utilise the software and programmes identified in the tool (e.g., Figure 1) – the specific design, construction, and implementation of this tool was unique and bespoke to a single UK youth male soccer academy possibly limiting generalisability to other TD environments with differing cultural, organisational, and sport-specific contexts. Secondly, although the tool incorporates longitudinal monitoring, empirical validation of the tool’s predictive validity and impact on TD and (de)selection remains to be established. Third, despite the integration of unbiased objective assessments in the form of signs and samples in addition to SEO’s, the subjectivity and potential for bias in TD and (de)selection cannot be eradicated. Instead, it should be noted that such a tool may facilitate and not replace these procedures, with a data-informed approach to decision-making potentially attenuating several possible (sub)conscious biases. Finally, implementing the tool in practice across other contexts may be prone to several barriers including access to appropriate technological infrastructure, staff training and engagement, opposition to implementation, and consistent data capture, all which may present challenges in resource-limited TD environments.

Future research may address several of these limitations by evaluating the tool and the recommended possible solutions in Parts 2 and 3 across different TD environments. Longitudinal studies would be recommended to validate the efficacy of the tool and to determine the extent to which TD and (de)selection decision-making is enhanced and improves developmental outcomes. Further, research could qualitatively assess the acceptance and value of the tool in relation to multidisciplinary decision-making providing further insight. Finally, adaptations for lower-resource environments, such as simplified or low-technology formats, should be explored to widen accessibility and practical impact across diverse TD contexts.

## Conclusion

In Part 1, real-world, practical challenges relating to recognising talent, evaluating performance, and athlete development were presented surrounding the need to better understand potential and performance, have an aligned and coherent approach, and to collect relevant multidisciplinary from various sources (signs, samples, and SEO) longitudinally. Part 2 presented possible solutions to these challenges and introduced the use of a comprehensive multidisciplinary athlete profiling tool incorporating these solutions within a youth male soccer academy in the UK. Further challenges and possible solutions to implementing such a tool were highlighted in Part 3, surrounding the processing and sharing of data from multiple sources, providing access to multiple stakeholders, and the appropriate use and engagement with the tool for TD and (de)selection purposes. Whilst limitations arise concerning the generalisability of findings from within a single UK soccer academy, many of the challenges and possible solutions presented are inherent across youth team sport talent systems. As such, the solutions presented and the applied tool may act as a guide, which can be interpreted and implemented flexibly across a variety of environments to improve (de)selection and TD processes.

## Data Availability

The original contributions presented in the study are included in the article/supplementary material, further inquiries can be directed to the corresponding author/s.
